# Blood and lung microRNAs as biomarkers of pulmonary tumorigenesis in cigarette smoke-exposed mice

**DOI:** 10.18632/oncotarget.12475

**Published:** 2016-10-05

**Authors:** Alberto Izzotti, Roumen Balansky, Gancho Ganchev, Marietta Iltcheva, Mariagrazia Longobardi, Alessandra Pulliero, Marta Geretto, Rosanna T. Micale, Sebastiano La Maestra, Mark Steven Miller, Vernon E. Steele, Silvio De Flora

**Affiliations:** ^1^ Department of Health Sciences, University of Genoa, Genoa, Italy; ^2^ IRCCS AOU San Martino IST, Genoa, Italy; ^3^ National Center of Oncology, Sofia, Bulgaria; ^4^ Chemopreventive Agent Development Research Group, Division of Cancer Prevention, National Cancer Institute, Bethesda, MD, USA

**Keywords:** lung microRNA, blood microRNA, lung carcinogenesis, cigarette smoke, intergender differences

## Abstract

Cigarette smoke (CS) is known to dysregulate microRNA expression profiles in the lungs of mice, rats, and humans, thereby modulating several pathways involved in lung carcinogenesis and other CS-related diseases. We designed a study aimed at evaluating (*a*) the expression of 1135 microRNAs in the lung of Swiss H mice exposed to mainstream CS during the first 4 months of life and thereafter kept in filtered air for an additional 3.5 months, (*b*) the relationship between lung microRNA profiles and histopathological alterations in the lung, (*c*) intergender differences in microRNA expression, and (*d*) the comparison with microRNA profiles in blood serum. CS caused multiple histopathological alterations in the lung, which were almost absent in sham-exposed mice. An extensive microRNA dysregulation was detected in the lung of CS-exposed mice. Modulation of microRNA profiles was specifically related to the histopathological picture, no effect being detected in lung fragments with non-neoplastic lung diseases (emphysema or alveolar epithelial hyperplasia), whereas a close association occurred with the presence and multiplicity of preneoplastic lesions (microadenomas) and benign lung tumors (adenomas). Three microRNAs regulating estrogen and HER2-dependent mechanisms were modulated in the lung of adenoma-bearing female mice. Blood microRNAs were also modulated in mice affected by early neoplastic lesions. However, there was a poor association between lung microRNAs and circulating microRNAs, which can be ascribed to an impaired release of mature microRNAs from the damaged lung. Studies in progress are evaluating the feasibility of analyzing blood microRNAs as a molecular tool for lung cancer secondary prevention.

## INTRODUCTION

Exposure to cigarette smoke (CS) has been shown to extensively dysregulate the expression of microRNAs (miRNAs), mostly in the sense of downregulation, in pulmonary cells of mice [1], rats [2], and humans [3]. CS-related downregulation of miRNAs translates into upregulation of gene and protein expression [4, 5]. These molecular processes result in the modulation both of adaptive mechanisms, protecting the organism from noxious CS components, and of mechanisms involved in the pulmonary carcinogenesis process, such as stress response, DNA repair, protein repair or removal, phagocytosis, endocytosis and intracellular vesicular traffic, immune response, cell proliferation, apoptosis, oncogene activation, inhibition of oncosuppressor genes, recruitment of undifferentiated stem cells, inflammation, inhibition of gap-junctional intercellular communications, and angiogenesis [4, 5].

In a preliminary study, we analyzed miRNA profiles in the lung of 2 sham-exposed mice, 3 mice exposed to mainstream CS (MCS), and 3 MCS-exposed mice treated with chemopreventive agents. The results suggested that miRNA expression was affected by histopathology, with specific signatures related to occurrence of pneumonia, adenoma, or bronchoalveolar carcinoma [6]. The present study had multiple goals. The first one was to comparatively evaluate miRNA expression profile in samples from sham-exposed mice and mice that had been exposed to MCS during the first 4 months of life and thereafter kept in filtered air for an additional 3.5 months. This carcinogenesis model has been developed in our laboratories [7] and applied to investigate both efficacy and safety of a number of dietary and pharmacological agents in CS-related carcinogenesis [8]. Both the whole lungs and the lung fragment undergoing miRNA analysis were subjected, as blind samples, to lung histopathology. This allowed us to evaluate pulmonary miRNA expression profiles as related to the occurrence of non-neoplastic lesions (lung emphysema), preneoplastic lesions (hyperplasia of the alveolar epithelium and microadenomas) and benign tumors (adenomas). In this way, it was possible to assess both sensitivity and specificity of miRNA analysis in characterizing these lesions. Such a methodological approach bears relevance because these alterations represent a still reversible step of the carcinogenesis process, thereby providing an ideal target for preventive interventions. Using both male and female mice, a second goal of the present study was to evaluate intergender differences in order to explore, from a mechanistic point of view, the suspected role of estrogens in CS-related pulmonary carcinogenesis [8]. Finally, by comparing miRNA profiles in the lung and blood serum of all mice, we aimed at validating the detection of circulating miRNAs as a secondary prevention tool to be used in translational studies and in possible applications in humans.

## RESULTS

### Lung histopathology

**Figure 1:**
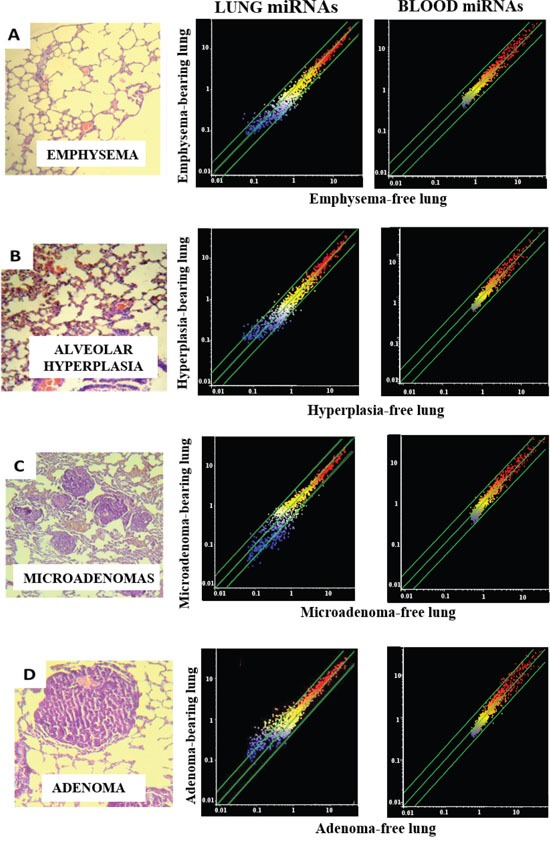
miRNA expression intensity in mouse lung and blood serum as related to pulmonary histopathological alterations The column on the left shows examples of microscopic appearance of pulmonary histopathological alterations, including emphysema **(A)**, alveolar epithelial hyperplasia **(B)**, microadenomas **(C)**, and adenomas **(D)**. The scatter-plots relate the expression of 1135 miRNAs, either in the lung (middle column) or in the blood serum (right column) of lesion-bearing mice to that in lesion-free mice. Each dot represents a miRNA, whose expression intensity can be inferred from the position on the x and y axes. The central diagonal lines indicate equivalence in the intensity of miRNA expression, and the outer diagonal lines indicate 2-fold differences in miRNA expression in lesion-bearing mice and lesion-free mice.

Table 1 reports the occurrence of emphysema, alveolar epithelial hyperplasia, microadenomas, and adenomas, as evaluated in 10 sham-exposed mice and 10 MCS-exposed mice by histopathological analysis either of the fragment of right caudal lobe used for miRNA analysis (A) or of the whole lungs from the same mice (B). The numbers in parentheses indicate the number of lesions detected in each mouse positive either for microadenomas or adenomas. Examples of the microscopic appearance of the above histopathological alterations are given in Figure 1.

**Table 1:** Histopathological alterations in the lung of Swiss H mice aged 7.5 months as related to exposure to MCS

Treatment	Gender	Identif. code	Emphysema		Alveolar epithelial hyperplasia		Microadenomas		Adenomas	
			A	B	A	B	A	B	A	B
Sham	Males	SM 2	-	-	-	-	-	-	-	-
		SM 4	-	-	-	-	-	-	-	-
		SM 5	-	-	-	-	-	-	-	-
		SM 14	-	-	-	-	-	-	-	-
		SM 15	-	-	-	-	-	-	-	-
	Females	SF 7	-	-	-	-	-	-	-	-
		SF 9	-	-	-	-	-	-	-	-
		SF 11	-	-	-	+	-	+ (3)	-	-
		SF 12	-	-	-	-	-	-	-	-
		SF 16	-	-	-	-	-	-	-	-
MCS	Males	MM 2	-	+	+	+	+ (6)	+ (6)	-	-
		MM 3	-	-	-	+	+ (8)	+ (25)	-	-
		MM 4	-	-	-	-	+ (4)	+ (25)	+ (1)	+ (1)
		MM 11	-	-	+	+	-	-	-	+ (7)
		MM 12	-	-	+	+	+(12)	+ (24)	-	-
	Females	MF 6	-	-	+	+	+(13)	+ (25)	-	-
		MF 7	-	-	-	+	+(5)	+ (5)	-	+ (10)
		MF 8	-	-	-	-	+(4)	+ (8)	-	+ (5)
		MF 9	+	+	+	+	+(6)	+ (21)	-	+ (6)
		MF 14	-	-	-	-	-	+ (8)	-	-

A. Fragment of right caudal lobe (1 section/mouse) used for miRNA analysis. B. Whole lungs (10 sections/mouse)

The numbers in parentheses indicated the total number of lesions (microadenomas or adenomas) per mouse. When microadenomas were confluent or were >25/mouse, a score of 25 was given.

The results show that none of the sham-exposed mice had histopathological lesions, with the exception of one female that developed 3 microadenomas detectable in the whole lungs. In contrast, a number of lung lesions were detected in MCS-exposed mice. Even in exposed mice, however, the incidence of emphysema was modest (20%), with positive findings in the whole lungs from a male and in both whole lungs and caudal lobe fragment from a female.

In contrast, the majority of mice (80% of males and 60% of females) exhibited signs of hyperplasia of the alveolar epithelium, mostly by analyzing the whole lungs (the 70% of MCS-exposed mice) but also by analyzing the caudal lobe fragment (50%). In both cases, the difference between sham-exposed mice and MCS-exposed mice was statistically significant (*P* < 0.01).

The large majority of the MCS-exposed mice (90%) developed multiple microadenomas, which in 3 mice became confluent by analyzing the whole lungs. Irrespective of gender, the difference in microadenoma incidence was statistically significant (*P* < 0.01) both in the caudal lobe fragment and in whole lungs. The multiplicity of microadenomas in the caudal lobe fragment from MCS-exposed mice (mean ± SE) was 6.0 ± 2.0 in males (*P* < 0.05 as compared with sham-exposed mice), 5.6 ± 2.1 in females (*P* < 0.05), and 5.8 ± 1.4 in combined genders (*P* < 0.001). In the corresponding whole lungs, the multiplicity of adenomas was 16.0 ± 5.4 in males (*P* < 0.05 as compared with sham-exposed mice, all of them microadenoma-free), 13.4 ± 4.0 in females (*P* < 0.05 *vs.* 0.6 ± 0.6 of sham-exposed mice), and 14.7 ± 3.2 in combined genders (*P* < 0.001 *vs.* 0.3 ± 0.3 of sham-exposed mice).

Adenomas were detected in the whole lungs from 2 males and 3 females and in the caudal lobe fragment from one male. As compared with sham-exposed mice, in which no adenoma was detected, the increase in the incidence of adenomas in whole lungs was statistically significant in females (*P* < 0.05) and in combined genders (*P* < 0.01). The increase in the multiplicity of adenomas in the caudal lobe fragment was not statistically significant. In whole lungs it was 1.6 ± 1.4 in males (not significant as compared to sham-exposed mice), 4.2 ± 1.9 in females (*P* < 0.05), and 2.9 ± 1.2 in combined genders (*P* < 0.05). The difference in adenoma multiplicity between males and females was not statistically significant.

### miRNA analyses in lung

Figure 1 (middle column) shows scatter-plots comparing the expression of miRNAs as related to occurrence of either emphysema (A), alveolar epithelial hyperplasia (B), microadenomas (C) or adenomas (D) in the caudal lobe fragment of lungs from all mice positive for the above lesions, as compared to mice negative for the same lesions. The diagonal lines indicate the ±2-fold variation interval in miRNA expression between lesion-bearing mice and lesion-free mice. Little or no alterations of miRNA expression were observed in mice bearing either emphysema (Figure 1A) or alveolar epithelial hyperplasia (Figure 1B) and, as demonstrated by volcano-plot analysis, none of them was statistically significant (data not shown).

**Figure 2:**
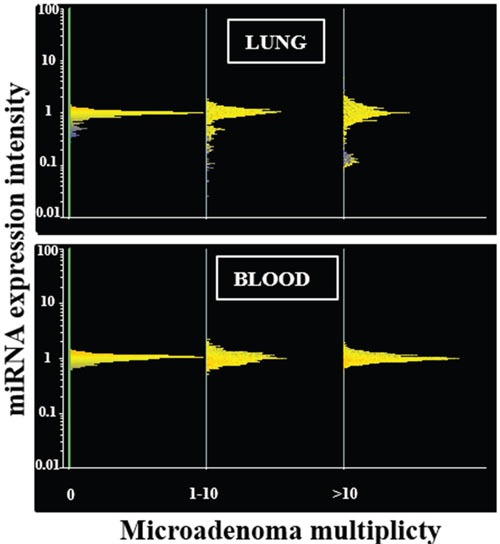
miRNA expression intensity in mouse lung and blood serum as related to the multiplicity of lung microadenomas Line-plot analyses show miRNA profiles in the lung (upper panel) and blood serum (lower panel) of mice as related to the multiplicity of lung microadenomas. miRNAs are distributed in horizontal lines according to their level of expression, the majority being located at intermediate levels of expression (central part of the distribution) and the minority being located at high and low levels of expression (lower and upper part of the distribution). The distribution profile is progressively modified according to the multiplicity of lung microadenomas in lung but not in blood.

In contrast, miRNA expression profiles were profoundly altered in those lung fragments, almost all of them from MCS-exposed mice, in which microadenomas were detected both in whole lungs and in the same tissue fragments in which the miRNA expression was analyzed. In particular, line-plot analyses (Figure 2) showed that the alterations of miRNA profile progressively increased with the multiplicity of microadenomas, and became massive when >10 microadenomas were present in the lung fragment. A relationship between microadenoma multiplicity and alterations of miRNA profiles was also evident from scatter-plot analysis (Figure 1C). According to volcano-plot analysis, 16 miRNAs altered their expression >2-fold and above the statistical significance threshold in mice bearing >10 microadenomas, as compared to microadenoma-free mice. Of these miRNAs, 12 were upregulated (miR-34b, miR-138, miR-297a, miR-301, miR-449, miR-466, miR-493, miR-579, miR-582, miR.-673, miR-692, and miR-879) and 4 were downregulated (miR-106a, miR-181a, miR-369, and miR-669k). The trend of alteration (up or downregulation), the fold-variation, and the biological functions of these miRNAs are reported in Table 2.

**Table 2:** List of miRNAs altered in lung or blood as related to the histopathological alteration detected in lung

miRNA	Microadenoma		Adenoma		Biological function
	Lung	Blood	Lung	Blood	
let-7a^§^			↑2.0		k-Ras inhibition [9]
let-7b			↓2.0	↑2.4	Inflammation, Angiogenesis [10]
let-7f			↓2.1		Proliferation, Apoptosis [11, 12]
miR-15a^§^			↓2.1		Proliferation, Inflammation, Apoptosis [13]
miR-21*			↓2.1		Proliferation, Apoptosis, Invasion [14–17]
miR-22			↑2.1	↑2.0	Proliferation, Invasion, Lipid folate catabolism [18–20]
miR-26b			↓5.0		Proliferation, Invasion [21–25]
miR-30^§^			↓4.1		Invasion, EMT [26–28]
miR-34b	↑4.9			↑2.2	P53 Effector, Proliferation, Apoptosis [29]
miR-106a	↓6.5			↑2.2	Proliferation, Apoptosis [30]
miR-124			↑2.1	↑2.0	Proliferation, Invasion, Apoptosis, Angiogenesis, EMT [31–34]
miR-125^°^			↑2.2	↑2.7	Proliferation, Invasion, *Erbb2* Suppression [35–40]
miR-129			↑2.5		Proliferation, Invasion, Apoptosis [41–43]
miR-138	↑2.1				Proliferation, Apoptosis, EMT [44–48]
miR-181a	↓8.4				Proliferation, Angiogenesis, EMT [49, 50]
miR-182^§^			↓4.2		Proliferation, Invasion, Differentiation, Ras inhibition [51]
miR-206			↑2.0	↑2.1	Proliferation, Invasion, Glycolysis suppression, EMT [52–56]
miR-208b			↓5.5		NA
miR-210			↓2.9		Proliferation, Apoptosis, Angiogensis [57]
miR-297a	↑2.2				Invasion [58]
miR-301	↑3.2		↑2.1	↑2.0	Invasion, Autophagy [59–61]
miR-326			↓2.4		Proliferation [62]
miR-339			↑2.1		Proliferation, Invasion, Tumor suppression [63–66]
miR-344			↓2.0	↓3.2	NA
miR-346			↓2.4		Proliferation [67, 68]
miR-362			↓2.3		Proliferation, Invasion, Apoptosis [69–76]
miR-369	↓2.8		↓2.6	↓2.1	Aerobic glycolysis [77]
miR-374			↑3.0	↓2.2	NA
miR-449	↑2.7			↑2.4	Proliferation [78–81]
miR-463			↓2.7		NA
miR-466^°^	↑2.4	↑2.1		↓3.5	NA
miR-483			↓3.2		Apoptosis [82]
miR-493	↑2.1			↓2.2	Proliferation [83–85]
miR-499a			↓5.0	↑2.3	Proliferation [86]
miR-504			↓2.6	↑2.0	Proliferation, Apoptosis [87, 88]
miR-579	↑2.8				NA
miR-582^^^	↑2.4				Proliferation [89]
miR-615			↓2.1		Proliferation, Invasion [90, 91]
miR-652		↑2.4			Proliferation, EMT [92, 93]
miR-669b			↓2.1		NA
miR-669h			↓3.6	↑2.3	NA
miR-669i			↓2.3		NA
miR-669k	↓7.2		↓5.8		NA
miR-673	↑2.1				NA
miR-692	↑2.1				NA
miR-762		↑2.2			Proliferation [94]
miR-767			↓2.3		DNA methylation [95]
miR-804^§^			↓2.0		Proliferation, Ras inhibition, Intercellular adhesion (Cx43) [6]
miR-879	↑2.2				NA
miR-1193			↑2.1		NA
miR-3080			↑3.5	↓2.9	NA

The numbers indicate the ratio of miRNA expression (fold-variation) between mice bearing either microadenomas and/or adenomas and lesions-free mice

NA, not available. EMT, Epithelial-Mesenchymal Transition.

§ Altered in CS induced lung adenocarcinoma [6]

° Downregulated in CS induced pneumonia [6]

^ Different between males and females in mice bearing >10 microadenomas.

**Figure 3:**
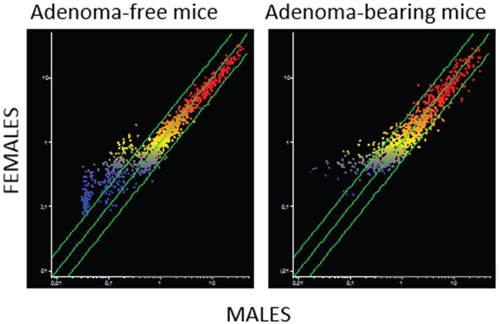
Intergender differences in miRNA expression intensity in mouse lung as related to the presence of pulmonary adenomas The scatter-plots relate the expression of 1135 pulmonary miRNAs in the lung of males to that in the lung of females, either adenoma-bearing or adenoma-free. Each dot represents a miRNA, whose expression intensity can be inferred from the position on the x and y axes. The central diagonal lines indicate equivalence in the intensity of miRNA expression, and the outer diagonal lines indicate 2-fold differences in miRNA expression in males and females.

A close relationship between alteration of miRNA profiles and presence of adenomas was also detected in lung fragments (Figure 1D). In particular, volcano-plot analyses showed that 36 miRNAs were significantly altered in mice bearing adenomas, all of them MCS-exposed, as compared to adenoma-free mice. Eleven miRNAs were upregulated and 25 were downregulated (see their identification in Table 2). Three dysregulated miRNAs (miR-301, miR-369, and miR-669k) overlapped in the lungs of adenoma-bearing mice and of microadenoma-bearing mice. In order to evaluate intergender differences, miRNA profiles were compared in males and females from either adenoma-free or adenoma-bearing mice. Scatter-plot analyses (Figure 3) provided evidence that miRNA profiles were influenced by the gender, although the differences mainly affected miRNAs that were expressed at low and intermediate intensity (blue and yellow colors). According to volcano-plot analyses, no miRNA was different in males and females from adenoma-free mice, whereas 3 miRNAs (miR-10a, miR-125, and miR- 130a) from adenoma-bearing mice showed intergender differences.

**Figure 4:**
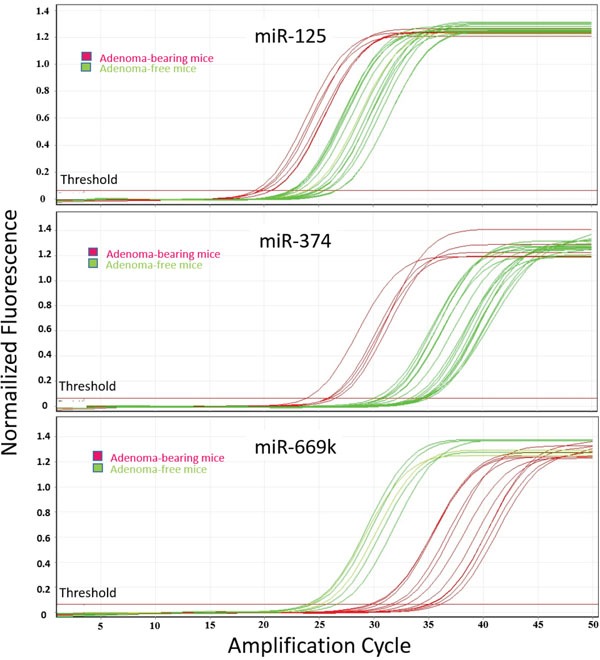
qPCR analysis of lung miRNAs The panels report the amplification curves for each one of the 20 mouse lung fragments tested, either adenoma-free (green) or adenoma-bearing (purple), relatively to miRNAs miR-125, miR-374, and miR-669k.

Validation of microarray data was performed by real time-qPCR for miR-125, miR-374, and miR-669k. The expression levels of these miRNAs were evaluated by testing each one of the 20 lung fragments, thus accounting for a total of 60 samples tested in triplicates. The results were related to the presence or absence of adenomas in the lung. Figure 4 reports the amplification curves for each sample, either adenoma-free (green) or adenoma-bearing (purple). The relative expression intensities of miR-125 were 2.8 ± 1.6 in adenoma-free mice and 5.6 ± 2.7 in adenoma-bearing mice, thus accounting for a 2.0-fold upregulation. This trend is similar to the 2.2-fold upregulation in adenoma-bearing mice detected by microarray (see Table 2). The relative expression intensities of miR-374 were 8.9 ± 2.4 in adenoma-free mice and 21.1 ± 6.9 in adenoma-bearing mice, thus accounting for a 2.4-fold upregulation, which is in line with the 3.0-fold upregulation detected in adenoma bearing mice by microarray (see Table 2). The relative expression intensities of miR-669k were 3.4 ± 1.3 in adenoma-free mice and 0.9 ± 0.2 in adenoma-bearing mice, thus accounting for a 3.5-fold downregulation, which is comparable to the 5.8-fold downregulation detected in adenoma-bearing mice by microarray (see Table 2). On the whole, qPCR results confirmed the trends observed by microarray for variations in miRNA expression as related to the presence of lung adenomas. However, the differences recorded by qPCR were less pronounced than those detected by the semi-quantitative microarray approach.

### MiRNA analyses in blood serum

Scatter-plot analyses (Figure 1, right column) provided evidence that miRNA levels in blood serum are not changed in mice affected either by emphysema (Figure 1A) or alveolar epithelium hyperplasia (Figure 1B). Likewise, circulating miRNAs were poorly affected by the presence of lung microadenomas, as shown both by scatter-plot analyses (Figure 1C) and line-plot analyses (Figure 2). According to volcano-plot analyses, only 3 miRNAs were significantly altered in the blood of mice bearing >10 microadenomas as compared to the other mice (Table 2). MiR-466 was the only miRNA that was upregulated both in lung and blood of mice bearing >10 microadenomas.

A more robust relationship was detected between presence of lung adenomas and alterations of miRNA profiles in blood serum (Figure 1D). In fact, a total of 18 blood miRNAs were significantly altered by the presence of lung adenomas, 12 of which were upregulated and 6 were downregulated (Table 2).

### Comparison of miRNAs in lung and blood serum

Venn diagram analyses (not shown) compared miRNA alterations in lung and blood serum as related to occurrence in the lung either of microadenomas or adenomas. One miRNA only (miR-466) was altered in both body compartments of mice bearing >10 microadenomas in the lung fragment. Conversely, 13 miRNAs were altered in both body compartments of mice bearing lung adenomas. The identity, fold-change variation, direction of alteration, and biological function of these miRNAs are reported in Table 2. In mice bearing adenomas, 5 miRNAs (miR-34b, miR-106a, miR-499, miR-466, and miR-493) were altered in the blood serum but not in lung.

## DISCUSSION

The results of histopathological analyses confirmed that exposure of mice to MCS during the first 4 months of life, followed by 3-4 months in filtered air in order to allow a better growth of pulmonary lesions, results in the appearance of significant alterations and especially of alveolar epithelial hyperplasias, microadenomas, and adenomas. While malignant lung tumors had been detected in broader groups of MCS-exposed mice [96], no such lesion was present in the subset of mice that was randomly selected for evaluating miRNA profiles. In any case, evaluation of MCS carcinogenicity was not the primary goal of the present study, the validity of the model used [7] having been already validated in a number of previous studies [reviewed in ref. 8]. Likewise, downregulation of miRNA expression in the lung of MCS-exposed mice and its modulation by chemopreventive agents have previously been established [1, 6, 97–100].

What differentiates the present study from the above cited studies, with one exception [6], is that so far we analyzed miRNAs in the lung of currently smoking mice, whereas here we evaluated miRNA profiles in both lung and blood serum of mice that had discontinued exposure to MCS 3.5 months earlier. Therefore, the observed changes in miRNA profiles may either reflect long-lasting alterations induced by MCS and/or the development of histopathological damage. The latter mechanism is clearly supported by the finding that miRNA alterations were specific and selective for microadenomas and adenomas, while no alteration was detected in the lung of mice affected either by emphysema and/or by alveolar epithelial hyperplasia. Microadenomas, which were consistently detected in all our studies using the carcinogenesis model in MCS-exposed mice [reviewed in ref. 8], are lesions larger than hyperplastic foci, some of which may progress further to adenomas, although they tend to regress spontaneously [101]. The membrane glycoprotein CD45, a leukocyte common antigen that is expressed in all haematopoietic cells, is not detectable in microadenomas [R. Balansky and G. Ganchev, unpublished data]. The fact that both lung microadenomas and adenomas were detected in the same mice supports the view that adenomas originates from microadenomas. This situation raises the question whether microadenomas are preneoplastic lesions or inflammatory lesions. This is important in order to explain the differences in miRNA profiles between lung tissue fragments containing microadenomas and adenomas. The data reported in this study indicate that overlap of miRNA expression changes in microadenoma and adenomas occur to a low extent. Indeed, only 3 miRNAs out of the 1135 examined (0.3%) were altered in both lesions. These miRNAs were miR-301, miR-369, and miR-669k, whose main functions are to regulate cell proliferation, autophagy, and aerobic glycolysis, which are mechanisms involved in the initial stage of the functional transformation of cells into their neoplastic counterpart. The finding that 33 miRNAs were altered in adenoma but not in microadenoma highlights the profound biological and molecular difference between these lesions. These 33 adenoma-specific miRNAs are involved in triggering the transition from microadenoma to full blown adenoma, thus playing an important role in tumor progression. These data indicate that miRNAs play a pivotal role in tumor progression and that microadenomas are different lesions from adenomas and not merely small-size adenomas.

Adenoma-related miRNA alterations were oriented both towards upregulation and downregulation. These patterns are different from those observed in malignant adenocarcinomas, in which miRNA downregulation prevails [6]. However, few miRNAs (4 out of the 1135 tested [0.3%]) were altered in both lesions. These miRNAs (miR-15a, miR-30, miR-182, and miR-804) are involved in cell proliferation, apoptosis, inflammation, epithelial-mesenchymal transition, invasion, oncogene inhibition, and intercellular adhesion. Alteration of these functions plays a role in driving the progression from benign lung tumors (adenomas) to malignant tumors (adenocarcinomas). Thus, miRNA profiling indicates that progression of microadenomas to adenomas and ultimately to adenocarcinomas occurs in a continuous fashion by accumulating new molecular alterations driving the carcinogenesis process.

The miRNAs altered by MCS exposure regulate biological functions playing a major role in the various steps of lung carcinogenesis, including expression of mutated oncogenes, removal of damaged cells by apoptosis, cell proliferation, tissue inflammation, epithelial-mesenchimal transition, glycolysis alteration, angiogenesis, and invasion. An important difference between benign and malignant lung lesions induced by MCS is the maintenance of let-7 homeostasis. The data obtained in the present study provide evidence that the let-7 family, whose a-f isoforms were spotted on the microarray used, was not altered in either microadenoma or adenoma. Conversely, the let-7 irreversible downregulation is a hallmark of malignant lung cancer, including adenocarcinoma in mice [6, 102] and nonsmall cell lung cancer (NSCLC) in humans [103]. This finding underlies the difference of the miRNA molecular fingerprint between benign and malignant neoplastic lesions in lung. Our previous studies demonstrated that an irreversible let-7 downregulation is a necessary step for MCS to display its full carcinogenic effect [98, 104]. Similarly, miR-34, an established p53 effector that is typically downregulated in malignant lung cancer [105], was upregulated in microadenomas but not in adenomas, as demonstrated in the present study. Thus, maintenance of miR-34 expression is a prerequisite to avoid the passage from benign to malignant cancer lesions in lung tissue.

A crucial issue in carcinogenesis is the occurrence of gender-specific mechanisms that may contribute to cancer susceptibility and development. The hypothesis that females may be more susceptible than males to CS-related lung carcinogenesis is still controversial in both humans [106] and mice [8]. Nevertheless, several experimental findings support the view that estrogens may contribute to the CS pulmonary carcinogenicity. For instance, studies in A/J mice showed the presence of 17β-estradiol in the lung and modulation of cytochrome P450 1b1 and other estrogen metabolism genes by CS [107]. Studies in heterozygous 129/SvJ Cyp1b1-KO mice suggested that CS accelerates the production of the 4-OHEs estrogen metabolites within the lung [108]. Moreover, the nonsteroidal anti-inflammatory drugs (NSAIDs) aspirin and naproxen, which are known to have antiestrogenic properties, selectively inhibited lung carcinogenesis in female mice exposed either to MCS [96] or to environmental CS [109]. In humans, a higher expression of CYP1A1 and levels of DNA adducts were found in the nontumorous lung tissue collected from female NSCLC patients [110]. Estrogens are functional in normal lung and tumor cell lines and can directly stimulate the transcription of estrogen-responsive genes in the nucleus of lung cells, thereby transactivating the epidermal growth factor pathway [111]. Our study showed that no miRNA was different between males and females in adenoma-free mice, while 3 miRNAs (miR-10a, miR-125, and miR-130a) were differentially expressed in adenoma-bearing male and female mice. In particular, miR-10a is related to estrogen dependent cancer promotion [112, 113], miR-130a both to the estrogen and HER2 pathways [114, 115], and miR-125 to HER2/erbb2 estrogen sensitive oncogene activation [116, 117]. These findings support the view that estrogen and HER2-dependent mechanisms can contribute to CS-induced lung carcinogenicity in females.

A major problem in cancer prevention is the use of minimally invasive sampling procedures testing surrogate blood fluids, such as blood, in order to obtain information regarding precancerous lesions in lung. Our results demonstrate that the occurrence of miRNA alterations in blood serum is only partial and is not directly related to lung expression profiles; miRNA alterations were not always the same in lung and blood. In fact, the directions of miRNA alterations coincided in blood and lung in 7 cases (5 upregulated and 2 downregulated miRNAs in both compartments), while they were divergent in 6 cases. This complex situation is likely to reflect the mechanism of release of miRNAs from the lung and the poor specificity of the blood miRNA pool. miRNA release from target organs is an active event driven by specific mechanisms, especially when occurring in organs exposed to carcinogens [118, 119]. Under these circumstances, the DICER enzyme is hit by electrophilic metabolites of carcinogens thereby blocking the maturation process to mature miRNAs [118]. Accordingly, the release of miRNA precursor is increased despite the downregulation of the corresponding mature miRNA, thus explaining the observed discrepancy between lung and blood miRNA in MCS-exposed mice developing adenomas. Conversely, for other miRNAs, the release from organs targeted by the carcinogenesis process is based on the extracellular release of mature miRNAs contained in microvesicles, which induce systemic effects in either neighbor or distant organs. As an example, this situation has been demonstrated for miR-29 released from cancer tissue and targeting skeletal muscle cells, which triggers cytopathic effect and cachexia [120]. The direct release of mature miRNAs is the likely mechanism of upregulation of miRNAs observed in both lung and blood in MCS-exposed mice bearing microadenomas or adenomas.

The differences in miRNA expression in lung and blood detected in our study also reflect the poor specificity of this body fluid, which can presumably be explained by the fact that CS is a systemic carcinogen. Indeed, miRNAs originating from multiple extra-pulmonary organs targeted by the genotoxic effects of cigarette smoke, such as liver, heart, kidney, and blood vessels, are released into the blood. In order to elucidate this issue, we have in progress a study that comparatively evaluates miRNA expression profiles in 10 organs and 3 body fluids of mice exposed to CS and/or treated with NSAIDs.

The data reported in the present study indicate that blood serum miRNAs may be used as biomarkers to detect the presence of still benign neoplastic lesions (adenomas) in lung, although blood miRNAs are by far less sensitive than lung miRNAs. However, the specificity of blood miRNAs in detecting lung adenomas is remarkable, since no alterations of blood miRNA profiles were detected in mice having either alveolar epithelial hyperplasia or lung emphysema.

Some of the miRNAs that we have found to be altered in either lung or blood of adenoma-bearing mice have been proposed for the early diagnosis of lung cancer in human trials analyzing peripheral blood. Such a situation occurred for miR-26b, miR-30, and miR-374 downregulation, and for miR-34, miR-301, and miR-352 upregulation [121]. Many of the miRNAs altered by MCS in mice, as shown in the present study, have also been found to be altered in the respiratory system of cancer-free smokers as compared to non-smokers [3], including miR-15, miR-30, miR-106, miR-125, miR-181, miR-362, and miR-652. These findings support the good translability of miRNA data obtained in experimental animal models to the human situation. Although differences in lung cancer susceptibility may even occur among different mouse strains [122], this circumstance supports supports the good translatability of miRNA results from mice to human, thanks to the high phylogenic stability of this molecular domain. However, as previously discussed, the patterns of miRNA alterations are remarkably different in benign and malignant lung cancer.

In conclusion, the results of the present study provide evidence that, even after a period of time from discontinuation of exposure to MCS, a number of miRNAs remain dysregulated in mouse lung. However, the alterations of miRNA profiles specifically occur in lung fragments containing preneoplastic lesions, such as microadenomas, and benign lesions, such as adenomas. Interestingly, miRNA alterations are gender-specific in adenoma-bearing lung fragments and involve modulation of miRNAs regulating estrogen-dependent mechanisms. The identification of circulating miRNAs, revealing the occurrence of early neoplastic lesions in lung, is particularly relevant for the secondary prevention of the most common cause of cancer mortality. Only a relatively small percentage of subjects undergoing exposure to environmental factors, such as CS, radon, or airborne pollution, develop lung cancer after long-term exposures. The identification of these high-risk subjects is a serious issue in setting up cancer screening programs. The use of circulating miRNA to identify subjects developing premalignant pulmonary lesions could represent a new tool to face these problems, thus providing an improvement in preventive strategy and targeted lung cancer screening.

## MATERIALS AND METHODS

### Design of the study

The present study used 20 strain H neonatal mice. Half of them (5 males and 5 females) were kept in filtered air for 7.5 months (sham-exposed mice). The other mice (5 males and 5 females) were exposed whole-body to MCS during the first 4 months of life, starting within 12 h after birth, and thereafter were kept in filtered air for an additional 3.5 months (MCS-exposed mice). These mice were randomized from larger groups of mice (65 sham-exposed mice and 69 MCS-exposed mice) that were used for evaluating the MCS-related genotoxic damage and histopathological alterations, along with the effects of NSAIDs [96]. We refer to the previous study for details on breeding and treatment of mice and for exposure conditions to MCS. At 7.5 months of life, all mice were euthanized by following the 2013 AVMA guidelines on euthanasia using slow introduction of CO_2_ asphyxiation. Death was confirmed by absence of respiration and/or heartbeat.

Blood was immediately collected by heart puncture and used for preparing serum. The whole lungs were collected, divided into 10 sections and used for histopathological analysis. In particular, the accessory, middle, and caudal lobes of the right lung were cut into two pieces each, whereas the cranial lobe was left uncut. The left lung was cut into 3 pieces.

A fragment of the right caudal lobe was divided into two parts, one fixed in 10% formalin and used for histopathological analysis (1 section/mouse), and the other one immersed in RNAlater (Qiagen, Valencia, CA) and used for miRNA analysis.

### miRNA extraction from lung and blood serum

For RNA extraction, the 40 lung fragments (10 mg each) were homogenized in QIAzol Lysis Reagent (700 μl) by continuous shaking in Tissue Lyser (Qiagen) for 2 min at 30 Hz. The homogenates were centrifuged at 14,000 x *g* at 4°C for 15 min to remove cell debris. Lung miRNA was purified from the supernatant by using a commercially available kit (miRNeasy, Qiagen). Blood serum miRNA was isolated by using the Exiqon's miRCURY™ RNA Isolation Kit – Biofluids (Exiqon, Vedbaek, Denmark).

The amount and purity of extracted RNA were evaluated by fiber optic spectrophotometer (Nanodrop ND-1000), and the 230/260 and 260/280 absorbance ratios were calculated. The RNA structural integrity was evaluated by capillary electrophoresis using a RNA bioanalyzer (Bioanalyzer Agilent 2100, Agilent Santa Clara, CA) equipped with a RNA oligonucleotide chip (RNA 6000 Nano Ladder Chip, Agilent). The miRNA amounts were accurately standardized among blood serum samples for microarray and qPCR analyses using Qubit™ 3.0 Fluorometer (Life Technologies, Gent, Belgium).

### miRNA expression analysis by microarray

miRNA expression was evaluated by miRCURY LNA™ microRNA Array (Exiqon), which contains 3100 capture probes covering human, mouse and rat miRNAs. In particular, this microarray analyzes the expression of 1135 mouse miRNAs. RNA from each sample was labeled with Label IT® miRNA Labeling Kits, Version 2 (Mirus Bio, WI) following the standard protocol. Total RNA (500 ng) was mixed with 10 μl of 10x labeling buffer, 4 μl Label IT reagent (containing Cy 3 or Cy 5 fluorescent tracers), and water to 86 μl. The samples were incubated at 36°C for 1 h and the reaction was stopped by adding 10 μl Stop Reagent. The samples were purified onto a chromatographic column, and hybridized to the microarray in GlassArray Hybridization Cassettes (Invitrogen Ltd, Paisley, UK) in a water bath at 37°C for 16 h, and then a wash sequence was performed. Microarray was dried by centrifugation and scanned by a laser scanner (ScanArray, PerkinElmer, Waltham, MA).

### miRNA analyses by qPCR

Validation of microarray data was performed by real time-qPCR for miR-125, miR-374, and miR-669k. SYBRGREEN fluorescent tracers was used to identify amplicons whose identity was checked by melting curve analysis according to previously published procedures [1]. Primer sequences (TIB Molbiol, Italy) were identified according to http://www.ncbi.nlm.nih.gov/tools/primer-blast/database. cDNAs were prepared using Superscript II Reverse Transcription kit (Invitrogen). PCR was performed in a Rotor-Gene 3000 Corbett Research, Mortlake, Australia). Each reaction was carried out using 10x PCR buffer, 50 mM MgCl_2_, dNTM mix, primerA 10 μM, primerS 10 μM, Platinum® Taq DNA polymerase (Invitrogen), cDNA (diluted 1:10), and SYBR GREEN^®^ (Invitrogen) in a 50-μL reaction volume. The thermal profile consisted of hot-start enzyme activation at 95°C for 2 minutes, 45 cycles of PCR at 94°C for 45 s (denaturation), gene-specific temperature annealing for 30 s, and 72°C for 30 s (elongation). Gene expression was normalized to the ribosomal subuint5 (r5S) housekeeping gene.

Each sample was tested in triplicate and the results were expressed as relative gene expression intensities as obtained from the first positive amplification cycle (Ct).

### Statistical analysis

The incidence of histopathological lesions was expressed as percent of mice affected by the lesions, and the statistical significance of the differences between groups was evaluated by χ^2^ analysis. The multiplicity of histopathological lesions was expressed as mean ± SE within each group of mice, and the statistical significance of the differences between groups was evaluated by ANOVA followed by Student's *t* test for unpaired data. *P* values lower than 0.05 were regarded as statistically significant.

Microarray data were log transformed, normalized, and analyzed by GeneSpring software (Agilent, Santa Clara, CA) after local background subtraction. Expression data were median centered by using the GeneSpring normalization option. Comparisons between sets of data were done by evaluating the fold variations of duplicate data generated for each miRNA. In addition, the statistical significance of the differences was evaluated by means of the GeneSpring ANOVA applied by using Bonferroni multiple testing correction. As inferred from volcano-plot analysis, differences between sets of data with *P* < 0.05 and >2-fold variations were taken as significant.

## References

[1] Izzotti A, Calin GA, Steele VE, Croce CM, De Flora S (2009). Relationships of microRNA expression in mouse lung with age and exposure to cigarette smoke and light. FASEB Journal.

[2] Izzotti A, Calin GA, Arrigo P, Steele VE, Croce CM, De Flora S (2009). Downregulation of microRNA expression in the lungs of rats exposed to cigarette smoke. FASEB Journal.

[3] Schembri F, Sridhar S, Perdomo C, Gustafson AM, Zhang X, Ergun A, Lu J, Liu G, Zhang X, Bowers J, Vaziri C, Ott K, Sensinger K (2009). MicroRNAs as modulators of smoking-induced gene expression changes in human airway epithelium. Proceedings of the National Academy of Sciences.

[4] Izzotti A, Bagnasco M, Cartiglia C, Longobardi M, Balansky RM, Merello A, Lubet RA, De Flora S (2005). Chemoprevention of genome, transcriptome, and proteome alterations induced by cigarette smoke in rat lung. European Journal of Cancer.

[5] De Flora S, Balansky R, D'Agostini F, Cartiglia C, Longobardi M, Steele VE, Izzotti A (2012). Smoke-induced microRNA and proteome alterations and their modulation by chemopreventive agents. International Journal of Cancer.

[6] Izzotti A, Larghero P, Balansky R, Pfeffer U, Steele VE, De Flora S (2011). Interplay between histopathological alterations, cigarette smoke and cancer chemopreventive agents in defining microRNA profiles in mouse lung. Mutation Research.

[7] Balansky R, Ganchev G, Iltcheva M, Steele VE, D'Agostini F, De Flora S (2007). Potent carcinogenicity of cigarette smoke in mice exposed early in life. Carcinogenesis.

[8] De Flora S, Ganchev G, Iltcheva M, La Maestra S, Micale R, Steele VE, Balansky R (2016). Pharmacological modulation of lung carcinogenesis in smokers: preclinical and clinical evidence. Trends in Pharmacological Sciences.

[9] Wang XR, Luo H, Li HL, Cao L, Wang XF, Yan W, Wang YY, Zhang JX, Jiang T, Kang CS, Liu N, You YP, Chinese Glioma Cooperative Group (CGCG) (2013). Overexpressed let-7a inhibits glioma cell malignancy by directly targeting K-ras, independently of PTEN. Neuro Oncology.

[10] Wang Z, Xu L, Hu Y, Huang Y, Zhang Y, Zheng X, Wang S, Wang Y, Yu Y, Zhang M, Yuan K, Min W (2016). miRNA let-7b modulates macrophage polarization and enhances tumor-associated macrophages to promote angiogenesis and mobility in prostate cancer. Scientific Reports.

[11] Li D, Chen L, Zhao W, Hao J, An R (2016). MicroRNA-let-7f-1 is induced by lycopene and inhibits cell proliferation and triggers apoptosis in prostate cancer. Molecular Medicine Reports.

[12] Yan S, Han X, Xue H, Zhang P, Guo X, Li T, Guo X, Yuan G, Deng L, Li G (2015). Let-7f Inhibits Glioma Cell Proliferation, Migration, and Invasion by Targeting Periostin. Journal of Cellular Biochemistry.

[13] Aqeilan RI, Calin GA, Croce CM (2010). miR-15a and miR-16-1 in cancer: discovery, function and future perspectives. Cell Death Differentiation.

[14] Wu YR, Qi HJ, Deng DF, Luo YY, Yang SL (2016). MicroRNA-21 promotes cell proliferation, migration, and resistance to apoptosis through PTEN/PI3K/AKT signaling pathway in esophageal cancer. Tumor Biology.

[15] Lin L, Tu HB, Wu L, Liu M, Jiang GN (2016). MicroRNA-21 Regulates Non-Small Cell Lung Cancer Cell Invasion and Chemo-Sensitivity through SMAD7. Cellular Physiology and Biochemistry.

[16] Sekar D, Krishnan R, Thirugnanasambantham K, Rajasekaran B, Islam VI, Sekar P (2016). Significance of microRNA 21 in gastric cancer. Clinics and Research in Hepatology and Gastroenterology.

[17] Báez-Vega PM, Vargas IM, Valiyeva F, Rosado JE, Roman A, Flores J, Marcos-Martínez MJ, Vivas-Mejía PE (2016). Targeting miR-21-3p inhibits proliferation and invasion of ovarian cancer cells. Oncotarget.

[18] Fan W, Huang J, Xiao H, Liang Z (2016). MicroRNA-22 is downregulated in clear cell renal cell carcinoma, and inhibits cell growth, migration and invasion by targeting PTEN. Molecular Medicine Reports.

[19] Zuo QF, Cao LY, Yu T, Gong L, Wang LN, Zhao YL, Xiao B, Zou QM (2015). MicroRNA-22 inhibits tumor growth and metastasis in gastric cancer by directly targeting MMP14 and Snail. Cell Death & Disease.

[20] Koufaris C, Valbuena GN, Pomyen Y, Tredwell GD, Nevedomskaya E, Lau CH, Yang T, Benito A, Ellis JK, Keun HC (2016). Systematic integration of molecular profiles identifies miR-22 as a regulator of lipid and folate metabolism in breast cancer cells. Oncogene.

[21] Tsai MM, Huang HW, Wang CS, Lee KF, Tsai CY, Lu PH, Chi HC, Lin YH, Kuo LM, Lin KH (2016). MicroRNA-26b inhibits tumor metastasis by targeting the KPNA2/c-jun pathway in human gastric cancer. Oncotarget.

[22] Wang Y, Sun B, Zhao X, Zhao N, Sun R, Zhu D, Zhang Y, Li Y, Gu Q, Dong X, Wang M, An J (2016). Twist1-related miR-26b-5p suppresses epithelial-mesenchymal transition, migration and invasion by targeting SMAD1 in hepatocellular carcinoma. Oncotarget.

[23] Li D, Wei Y, Wang D, Gao H, Liu K (2016). MicroRNA-26b suppresses the metastasis of non-small cell lung cancer by targeting MIEN1 via NF-κB/MMP-9/VEGF pathways. Biochemical and Biophysical Research Communications.

[24] Xia M, Duan ML, Tong JH, Xu JG (2015). MiR-26b suppresses tumor cell proliferation, migration and invasion by directly targeting COX-2 in lung cancer. European Review for Medical and Pharmacological Sciences.

[25] Zheng WD, Zhou FL, Lin N (2015). MicroRNA-26b inhibits osteosarcoma cell migration and invasion by down-regulating PFKFB3 expression. Genetics and molecular research.

[26] Tsukasa K, Ding Q, Miyazaki Y, Matsubara S, Natsugoe S, Takao S (2016). miR-30 family promotes migratory and invasive abilities in CD133(+) pancreatic cancer stem-like cells. Human Cell.

[27] Li W, Liu C, Zhao C, Zhai L, Lv S (2016). Downregulation of β3 integrin by miR-30a-5p modulates cell adhesion and invasion by interrupting Erk/Ets-1 network in triple-negative breast cancer. International Journal of Oncology.

[28] Ye Z, Zhao L, Li J, Chen W, Li X (2015). miR-30d Blocked Transforming Growth Factor β1-Induced Epithelial-Mesenchymal Transition by Targeting Snail in Ovarian Cancer Cells. International Journal of Gynecological Cancer.

[29] Liu X, Feng J, Tang L, Liao L, Xu Q, Zhu S (2015). The regulation and function of miR-21-FOXO3a-miR 34b/c signaling in breast cancer. International Journal of Molecular Sciences.

[30] Zhu M, Zhang N, He S, Yan R, Zhang J (2016). MicroRNA-106a functions as an oncogene in human gastric cancer and contributes to proliferation and metastasis in vitro and in vivo. Clinical and Experimental Metastasis.

[31] Jiang CF, Li DM, Shi ZM, Wang L, Liu MM, Ge X, Liu X, Qian YC, Wen YY, Zhen LL, Lin J, Liu LZ, Jiang BH (2016). Estrogen regulates miRNA expression: implication of estrogen receptor and miR-124/AKT2 in tumor growth and angiogenesis. Oncotarget.

[32] Zhou L, Xu Z, Ren X, Chen K, Xin S (2016). MicroRNA-124 (MiR-124) Inhibits Cell Proliferation, Metastasis and Invasion in Colorectal Cancer by Downregulating Rho-Associated Protein Kinase 1(ROCK1). Cellular Physiology and Biochemistry.

[33] Deng D, Wang L, Chen Y, Li B, Xue L, Shao N, Wang Q, Xia X, Yang Y, Zhi F (2016). miR-124-3p regulates cell proliferation, invasion, apoptosis and bioenergetics by targeting PIM1 in astrocytoma. Cancer Science.

[34] Ma T, Zhao Y, Wei K, Yao G, Pan C, Liu B, Xia Y, He Z, Qi X, Li Z, Wang J, Shao Y (2016). MicroRNA-124 Functions as a Tumor Suppressor by Regulating CDH2 and Epithelial-Mesenchymal Transition in Non-Small Cell Lung Cancer. Cellular Physiology and Biochemistry.

[35] Yang J, Li G, Zhang K (2016). MiR-125a regulates ovarian cancer proliferation and invasion by repressing GALNT14 expression. Biomedicine & Pharmacotherapy.

[36] Zhang Y, Xue C, Zhu X, Zhu X, Xian H, Huang Z (2016). Suppression of microRNA-125a-5p upregulates the TAZ-EGFR signaling pathway and promotes retinoblastoma proliferation. Cellular Signalling.

[37] Chang S, He S, Qiu G, Lu J, Wang J, Liu J, Fan L, Zhao W, Che X (2016). MicroRNA-125b promotes invasion and metastasis of gastric cancer by targeting STARD13 and NEU1. Tumour Biology.

[38] Fan Z, Cui H, Xu X, Lin Z, Zhang X, Kang L, Han B, Meng J, Yan Z, Yan X, Jiao S (2015). MiR-125a suppresses tumor growth, invasion and metastasis in cervical cancerby targeting STAT3. Oncotarget.

[39] Qin X, Wan Y, Wang S, Xue M (2015). MicroRNA-125a-5p modulates human cervical carcinoma proliferation and migration by targeting ABL2. Journal of Drug Design, Development and Therapy.

[40] Zhao X, He W, Li J, Huang S, Wan X, Luo H, Wu D (2015). MiRNA-125b inhibits proliferation and migration by targeting SphK1 in bladder cancer. American Journal of Translational Research.

[41] Liu Z, Dou C, Yao B, Xu M, Ding L, Wang Y, Jia Y, Li Q, Zhang H, Tu K, Song T, Liu Q (2016). Methylation-mediated repression of microRNA-129-2 suppresses cell aggressiveness by inhibiting high mobility group box 1 in human hepatocellular carcinoma. Oncotarget.

[42] Tang X, Tang J, Liu X, Zeng L, Cheng C, Luo Y, Li L, Qin SL, Sang Y, Deng LM, Lv XB (2016). Downregulation of miR-129-2 by promoter hypermethylation regulates breast cancer cell proliferation and apoptosis. Oncology Reports.

[43] Kouhkan F, Mobarra N, Soufi-Zomorrod M, Keramati F, Hosseini Rad SM, Fathi-Roudsari M, Tavakoli R, Hajarizadeh A, Ziaei S, Lahmi R, Hanif H, Soleimani M (2016). MicroRNA-129-1 acts as tumour suppressor and induces cell cycle arrest of GBM cancer cells through targeting IGF2BP3 and MAPK1. Journal of Medical Genetics.

[44] Jiang B, Mu W, Wang J, Lu J, Jiang S, Li L, Xu H, Tian H (2016). MicroRNA-138 functions as a tumor suppressor in osteosarcoma by targeting differentiated embryonic chondrocyte gene 2. Journal of Experimental & Clinical Cancer Research.

[45] Li B, Yang XX, Wang D, Ji HK (2016). MicroRNA-138 inhibits proliferation of cervical cancer cells by targeting c-Met. European Review for Medical and Pharmacological Sciences.

[46] Jin Z, Guan L, Song Y, Xiang GM, Chen SX, Gao B (2016). MicroRNA-138 regulates chemoresistance in human non-small cell lung cancer via epithelial mesenchymal transition. European Review for Medical and Pharmacological Sciences.

[47] Zhang J, Liu D, Feng Z, Mao J, Zhang C, Lu Y, Li J, Zhang Q, Li Q, Li L (2016). MicroRNA-138 modulates metastasis and EMT in breast cancer cells by targeting vimentin. Biomedicine & Pharmacotherapy.

[48] Sun DK, Wang JM, Zhang P, Wang YQ (2015). MicroRNA-138 Regulates Metastatic Potential of Bladder Cancer Through ZEB2. Cellular Physiology and Biochemistry.

[49] Li Y, Kuscu C, Banach A, Zhang Q, Pulkoski-Gross A, Kim D, Liu J, Roth E, Li E, Shroyer KR, Denoya PI, Zhu X, Chen L (2015). miR-181a-5p inhibits cancer cell migration and angiogenesis via downregulation of matrix metalloproteinase-14. Cancer Research.

[50] Ma Z, Qiu X, Wang D, Li Y, Zhang B, Yuan T, Wei J, Zhao B, Zhao X, Lou J, Jin Y, Jin Y (2015). MiR-181a-5p inhibits cell proliferation and migration by targeting Kras in non-small cell lung cancer A549 cells. Acta Biochimica et Biophysica Sinica (Shanghai).

[51] Yao J, Xu C, Fang Z, Li Y, Liu H, Wang Y, Xu C, Sun Y (2016). Androgen receptor regulated microRNA miR-182-5p promotes prostate cancer progression by targeting the ARRDC3/ITGB4 pathway. Biochemical and Biophysical Research Communications.

[52] Yin K, Yin W, Wang Y, Zhou L, Liu Y, Yang G, Wang J, Lu J (2016). MiR-206 suppresses epithelial mesenchymal transition by targeting TGF-β signaling in estrogen receptor positive breast cancer cells. Oncotarget.

[53] Chen QY, Jiao DM, Wang J, Hu H, Tang X, Chen J, Mou H, Lu W (2016). miR-206 regulates cisplatin resistance and EMT in human lung adenocarcinoma cells partly by targeting MET. Oncotarget.

[54] Chen QY, Jiao DM, Wu YQ, Chen J, Wang J, Tang XL, Mou H, Hu HZ, Song J, Yan J, Wu LJ, Chen J, Wang Z (2016). MiR-206 inhibits HGF-induced epithelial-mesenchymal transition and angiogenesis in non-small cell lung cancer via c-Met /PI3k/Akt/mTOR pathway. Oncotarget.

[55] Xiao H, Xiao W, Cao J, Li H, Guan W, Guo X, Chen K, Zheng T, Ye Z, Wang J, Xu H (2016). miR-206 functions as a novel cell cycle regulator and tumor suppressor in clear-cell renal cell carcinoma. Cancer Letters.

[56] Ge X, Lyu P, Cao Z, Li J, Guo G, Xia W, Gu Y (2015). Overexpression of miR-206 suppresses glycolysis, proliferation and migration in breast cancer cells via PFKFB3 targeting. Biochemical and Biophysical Research Communications.

[57] Zhu W, Zhou K, Zha Y, Chen D, He J, Ma H, Liu X, Le H, Zhang Y (2016). Diagnostic Value of Serum miR-182, miR-183, miR-210, and miR-126 Levels in Patients with Early-Stage Non-Small Cell Lung Cancer. PLoS One.

[58] Kefas B, Floyd DH, Comeau L, Frisbee A, Dominguez C, Dipierro CG, Guessous F, Abounader R, Purow B (2013). A miR-297/hypoxia/DGK-α axis regulating glioblastoma survival. Neuro Oncology.

[59] Egawa H, Jingushi K, Hirono T, Ueda Y, Kitae K, Nakata W, Fujita K, Uemura M, Nonomura N, Tsujikawa K (2016). The miR-130 family promotes cell migration and invasion in bladder cancer through FAK and Akt phosphorylation by regulating PTEN. Scientific Reports.

[60] Guo YJ, Liu JX, Guan YW (2016). Hypoxia induced upregulation of miR-301a/b contributes to increased cell autophagy and viability of prostate cancer cells by targeting NDRG2. European Review for Medical and Pharmacological Sciences.

[61] Funamizu N, Lacy CR, Parpart ST, Takai A, Hiyoshi Y, Yanaga K (2014). MicroRNA-301b promotes cell invasiveness through targeting TP63 in pancreatic carcinoma cells. International Journal of Oncology.

[62] Wang R, Chen X, Xu T, Xia R, Han L, Chen W, De W, Shu Y (2016). MiR-326 regulates cell proliferation and migration in lung cancer by targeting phox2a and is regulated by HOTAIR. American Journal of Cancer Research.

[63] Weber CE, Luo C, Hotz-Wagenblatt A, Gardyan A, Kordaß T, Holland-Letz T, Osen W, Eichmuller SB (2016). miR-339-3p is a tumor suppressor in melanoma. Cancer Research.

[64] Zhou C, Lu Y, Li X (2015). miR-339-3p inhibits proliferation and metastasis of colorectal cancer. Oncology Letters.

[65] Shan W, Li J, Bai Y, Lu X (2016). miR-339-5p inhibits migration and invasion in ovarian cancer cell lines by targeting NACC1 and BCL6. Tumour Biology.

[66] Wang YL, Chen CM, Wang XM, Wang L (2016). Effects of miR-339-5p on invasion and prognosis of hepatocellular carcinoma. Clinics and Research in Hepatology and Gastroenterology.

[67] Chen B, Pan W, Lin X, Hu Z, Jin Y, Chen H, Ma G, Qiu Y, Chang L, Hua C, Zou Y, Gao Y, Ying H (2016). MicroRNA-346 functions as an oncogene in cutaneous squamous cell carcinoma. Tumour Biology.

[68] Du L, Borkowski R, Zhao Z, Ma X, Yu X, Xie XJ, Pertsemlidis A (2013). A high-throughput screen identifies miRNA inhibitors regulating lung cancer cell survival and response to paclitaxel. RNA Biology.

[69] Ni F, Gui Z, Guo Q, Hu Z, Wang X, Chen D, Wang S (2016). Downregulation of miR-362-5p inhibits proliferation, migration and invasion of human breast cancer MCF7 cells. Oncology Letters.

[70] Zou X, Zhong J, Li J, Su Z, Chen Y, Deng W, Li Y, Lu S, Lin Y, Luo L, Li Z, Cai Z, Tang A (2016). miR-362-3p targets nemo-like kinase and functions as a tumor suppressor in renal cancer cells. Molecular Medicine Reports.

[71] Kang H, Kim C, Lee H, Rho JG, Seo JW, Nam JW, Song WK, Nam SW, Kim W, Lee EK (2016). Downregulation of microRNA-362-3p and microRNA-329 promotes tumor progression in human breast cancer. Cell Death & Differentiation.

[72] Wu K, Yang L, Chen J, Zhao H, Wang J, Xu S, Huang Z (2015). miR-362-5p inhibits proliferation and migration of neuroblastoma cells by targeting phosphatidylinositol 3-kinase-C2β. FEBS Letters.

[73] Shen H, Li W, Tian Y, Xu P, Wang H, Zhang J, Li Y (2015). Upregulation of miR-362-3p Modulates Proliferation and Anchorage-Independent Growth by Directly TargetingTob2 in Hepatocellular Carcinoma. Journal of Cellular Biochemistry.

[74] Ni F, Zhao H, Cui H, Wu Z, Chen L, Hu Z, Guo C, Liu Y, Chen Z, Wang X, Chen D, Wei H, Wang S (2015). MicroRNA-362-5p promotes tumor growth and metastasis by targeting CYLD in hepatocellular carcinoma. Cancer Lett.

[75] Xia JT, Chen LZ, Jian WH, Wang KB, Yang YZ, He WL, He YL, Chen D, Li W (2014). MicroRNA-362 induces cell proliferation and apoptosis resistance in gastric cancer by activation of NF-κB signaling. J Transl Med.

[76] Christensen LL, Tobiasen H, Holm A, Schepeler T, Ostenfeld MS, Thorsen K, Rasmussen MH, Birkenkamp-Demtroeder K, Sieber OM, Gibbs P, Lubinski J, Lamy P (2013). MiRNA-362-3p induces cell cycle arrest through targeting of E2F1, USF2 and PTPN1 and is associated with recurrence of colorectal cancer. International Journal of Cancer.

[77] Wang J, Wang H, Liu A, Fang C, Hao J, Wang Z (2015). Lactate dehydrogenase A negatively regulated by miRNAs promotes aerobic glycolysis and is increased in colorectal cancer. Oncotarget.

[78] Yuan JM, Shi XJ, Sun P, Liu JX, Wang W, Li M, Ling FY (2015). Downregulation of cell cycle-related proteins in ovarian cancer line and cell cycle arrest induced by microRNA. International Journal of Clinical and Experimental Medicine.

[79] Zhang H, Feng Z, Huang R, Xia Z, Xiang G, Zhang J (2014). MicroRNA-449 suppresses proliferation of hepatoma cell lines through blockade lipid metabolic pathway related to SIRT1. International Journal of Clinical and Experimental Medicine.

[80] Fang Y, Gu X, Li Z, Xiang J, Chen Z (2013). miR-449b inhibits the proliferation of SW1116 colon cancer stem cells through downregulation of CCND1 and E2F3 expression. Oncology Reports.

[81] Luo W, Huang B, Li Z, Li H, Sun L, Zhang Q, Qiu X, Wang E (2013). MicroRNA-449a is downregulated in non-small cell lung cancer and inhibits migration and invasion by targeting c-Met. PLoS One.

[82] Lupini L, Pepe F, Ferracin M, Braconi C, Callegari E, Pagotto S, Spizzo R, Zagatti B, Lanuti P, Fornari F, Ghasemi R, Mariani-Costantini R, Bolondi L (2016). Over-expression of the miR-483-3p overcomes the miR-145/TP53 pro-apoptotic loop in hepatocellular carcinoma. Oncotarget.

[83] Zhou W, Zhang C, Jiang H, Zhang Z, Xie L, He X (2015). MiR-493 suppresses the proliferation and invasion of gastric cancer cells by targeting RhoC. Iranian Journal of Basic Medical Sciences.

[84] Jia X, Li N, Peng C, Deng Y, Wang J, Deng M, Lu M, Yin J, Zheng G, Liu H, He Z (2016). miR-493 mediated DKK1 down-regulation confers proliferation, invasion and chemo-resistance in gastric cancer cells. Oncotarget.

[85] Gu Y, Cheng Y, Song Y, Zhang Z, Deng M, Wang C, Zheng G, He Z (2014). MicroRNA-493 suppresses tumor growth, invasion and metastasis of lung cancer by regulating E2F1. PLoS One.

[86] Li M, Zhang S, Wu N, Wu L, Wang C, Lin Y (2016). Overexpression of miR-499-5p inhibits non-small cell lung cancer proliferation and metastasis by targeting VAV3. Scientific Reports.

[87] Cui R, Guan Y, Sun C, Chen L, Bao Y, Li G, Qiu B, Meng X, Pang C, Wang Y (2016). A tumor-suppressive microRNA, miR-504, inhibits cell proliferation and promotes apoptosis by targeting FOXP1 in human glioma. Cancer Letters.

[88] Kikkawa N, Kinoshita T, Nohata N, Hanazawa T, Yamamoto N, Fukumoto I, Chiyomaru T, Enokida H, Nakagawa M, Okamoto Y, Seki N (2014). microRNA-504 inhibits cancer cell proliferation via targeting CDK6 in hypopharyngeal squamous cell carcinoma. International Journal of Oncology.

[89] Uchino K, Takeshita F, Takahashi RU, Kosaka N, Fujiwara K, Naruoka H, Sonoke S, Yano J, Sasaki H, Nozawa S, Yoshiike M, Kitajima K, Chikaraishi T (2013). Therapeutic effects of microRNA-582-5p and -3p on the inhibition of bladder cancer progression. Molecular Therapy.

[90] Wu X, Deng L, Tang D, Ying G, Yao X, Liu F, Liang G (2016). miR-615-5p prevents proliferation and migration through negatively regulating serine hydromethyltransferase 2 (SHMT2) in hepatocellular carcinoma. Tumor Biology.

[91] Bai Y, Li J, Li J, Liu Y, Zhang B (2015). MiR-615 inhibited cell proliferation and cell cycle of human breast cancer cells by suppressing of AKT2 expression. International Journal of Clinical and Experimental Medicine.

[92] Yang W, Zhou C, Luo M, Shi X, Li Y, Sun Z, Zhou F, Chen Z, He J (2016). MiR-652-3p is upregulated in non-small cell lung cancer and promotes proliferation and metastasis by directly targeting Lgl1. Oncotarget.

[93] Deng S, Li X, Niu Y, Zhu S, Jin Y, Deng S, Chen J, Liu Y, He C, Yin T, Yang Z, Tao J, Xiong J (2015). MiR-652 inhibits acidic microenvironment-induced epithelial-mesenchymal transition of pancreatic cancer cells by targeting ZEB1. Oncotarget.

[94] Li Y, Huang R, Wang L, Hao J, Zhang Q, Ling R, Yun J (2015). microRNA-762 promotes breast cancer cell proliferation and invasion by targeting IRF7 expression. Cell Proliferation.

[95] Loriot A, Van Tongelen A, Blanco J, Klaessens S, Cannuyer J, van Baren N, Decottignies A, De Smet C (2014). A novel cancer-germline transcript carrying pro-metastatic miR-105 and TET-targeting miR-767 induced by DNA hypomethylation in tumors. Epigenetics.

[96] Balansky R, Ganchev G, Iltcheva M, Micale RT, La Maestra S, D'Oria C, Steele VE, De Flora S (2016). Selective inhibition by aspirin and naproxen of mainstream cigarette smoke-induced genotoxicity and lung tumors in female mice. Archives of Toxicology.

[97] Izzotti A, Larghero P, Cartiglia C, Longobardi M, Pfeffer U, Steele VE, De Flora S (2010). Modulation of microRNA expression by budesonide, phenethyl isothiocyanate, and cigarette smoke in mouse liver and lung. Carcinogenesis.

[98] Izzotti A, Larghero P, Longobardi M, Cartiglia C, Camoirano A, Steele VE, De Flora S (2011). Dose-responsiveness and persistence of microRNA expression alterations induced by cigarette smoke in mouse lung. Mutation Research.

[99] Izzotti A, Balansky R, D'Agostini F, Longobardi M, Cartiglia C, La Maestra S, Micale RT, Camoirano A, Ganchev G, Iltcheva M, Steele VE, De Flora S (2013). Relationships between pulmonary microRNA and proteome profiles, systemic cytogenetic damage, and lung tumors in cigarette smoke-exposed mice treated with chemopreventive agents. Carcinogenesis.

[100] Izzotti A, Balansky R, D'Agostini F, Longobardi M, Cartiglia C, Micale RT, La Maestra S, Camoirano A, Ganchev G, Iltcheva M, Steele VE, De Flora S (2014). Modulation by metformin of molecular and histopathological alterations in the lung of cigarette smoke-exposed mice. Cancer Medicine.

[101] O'Donnell EP, Zerbe LK, Dwyer-Nield LD, Kisley LR, Malkinson AM (2006). Quantitative analysis of early chemically-induced pulmonary lesions in mice of varying susceptibilities to lung tumorigenesis. Cancer Letters.

[102] Esquela-Kerscher A, Trang P, Wiggins JF, Patrawala L, Cheng A, Ford L, Weidhaas JB, Brown D, Bader AG, Slack FJ (2008). The let-7 microRNA reduces tumor growth in mouse models of lung cancer. Cell Cycle.

[103] Alam M, Ahmad R, Rajabi H, Kufe D (2015). MUC1-C Induces the LIN28B→LET-7→HMGA2 Axis to Regulate Self-Renewal in NSCLC. Molecular Cancer Research.

[104] Izzotti A, Pulliero A (2015). Molecular damage and lung tumors in cigarette smoke-exposed mice. Annals of the New York Academy of Sciences.

[105] Stahlhut C, Slack FJ (2015). Combinatorial action of microRNAs let-7 and miR-34 effectively synergizes with erlotinib to suppress non-small cell lung cancer cell proliferation. Cell Cycle.

[106] Kreuzer M, Boffetta P, Whitley E, Ahrens W, Gaborieau V, Heinrich J, Jöckel KH, Kreienbrock L, Mallone S, Merletti F, Roesch F, Zambon P, Simonato L (2000). Gender differences in lung cancer risk by smoking: a multicentre case-control study in Germany and Italy. British Journal of Cancer.

[107] Meireles SI, Esteves GH, Hirata R, Peri S, Devarajan K, Slifker M, Mosier SL, Peng J, Vadhanam MV, Hurst HE, Neves EJ, Reis LF, Gairola CG (2010). Early changes in gene expression induced by tobacco smoke: evidence for the importance of estrogen within lung tissue. Cancer Prevention Research.

[108] Peng J, Xu X, Mace BE, Vanderveer LA, Workman LR, Slifker MJ, Sullivan PM, Veenstra TD, Clapper ML (2013). Estrogen metabolism within the lung and its modulation by tobacco smoke. Carcinogenesis.

[109] La Maestra S, D'Agostini F, Izzotti A, Micale RT, Mastracci L, Camoirano A, Balansky R, Trosko JE, Steele VE, De Flora S (2015). Modulation by aspirin and naproxen of nucleotide alterations and tumors in the lung of mice exposed to environmental cigarette smoke since birth. Carcinogenesis.

[110] Mollerup S, Berge G, Baera R, Skaug V, Hewer A, Phillips DH, Stangeland L, Haugen A (2006). Sex differences in risk of lung cancer: expression of genes in the PAH bioactivation pathway in relation to smoking and bulky DNA adducts. International Journal of Cancer.

[111] Stabile LP, Siegfried JM (2004). Estrogen receptor pathways in lung cancer. Current Oncology Reports.

[112] Hoppe R, Achinger-Kawecka J, Winter S, Fritz P, Lo WY, Schroth W, Brauch H (2013). Increased expression of miR-126 and miR-10a predict prolonged relapse-free time of primary oestrogen receptor-positive breast cancer following tamoxifen treatment. European Journal of Cancer.

[113] Stadthagen G, Tehler D, Høyland-Kroghsbo NM, Wen J, Krogh A, Jensen KT, Santoni-Rugiu E, Engelholm LH, Lund AH (2013). Loss of miR-10a activates lpo and collaborates with activated Wnt signaling in inducing intestinal neoplasia in female mice. PLoS Genetics.

[114] Tang L, Pu Y, Wong DK, Liu T, Tang H, Xiang T, Yuen MF, Ren G (2011). The hepatitis B virus-associated estrogen receptor alpha (ERalpha) was regulated by microRNA-130a in HepG2. 2.15 human hepatocellular carcinoma cells. Acta Biochimica Biophysica Sinica (Shanghai).

[115] Stückrath I, Rack B, Janni W, Jäger B, Pantel K, Schwarzenbach H (2015). Aberrant plasma levels of circulating miR-16, miR-107, miR-130a and miR-146a are associated with lymph node metastasis and receptor status of breast cancer patients. Oncotarget.

[116] Yagishita S, Fujita Y, Kitazono S, Ko R, Nakadate Y, Sawada T, Kitamura Y, Shimoyama T, Maeda Y, Takahashi F, Takahashi K, Tamura T, Koizumi F (2015). Chemotherapy-Regulated microRNA-125-HER2 Pathway as a Novel Therapeutic Target for Trastuzumab-Mediated Cellular Cytotoxicity in Small Cell Lung Cancer. Molecular Cancer Therapeutics.

[117] Tang XY, Zheng W, Ding M, Guo KJ, Yuan F, Feng H, Deng B, Sun W, Hou Y, Gao L (2016). miR-125b acts as a tumor suppressor in chondrosarcoma cells by the sensitization to doxorubicin through direct targeting the ErbB2-regulated glucose metabolism. Drug Design, Development and Therapy.

[118] Izzotti A, Pulliero A (2014). The effects of environmental chemical carcinogens on the microRNA machinery. International Journal of Hygiene and Environmental Health.

[119] Ceccaroli C, Pulliero A, Geretto M, Izzotti A (2015). Molecular fingerprints of environmental carcinogens in human cancer. Journal of Environmental Science and Health Part C Environmental Carcinogenesis & Ecotoxicology Reviews.

[120] He WA, Calore F, Londhe P, Canella A, Guttridge DC, Croce CM (2014). Microvesicles containing miRNAs promote muscle cell death in cancer cachexia via TLR7. Proceedings of the National Academy of Sciences.

[121] Izzotti A, Carozzo S, Pulliero A, Zhabayeva D, Ravetti JL, Bersimbaev R (2016). Extracellular microRNA in liquid biopsy: applicability in cancer diagnosis and prevention. American Journal of Cancer Research.

[122] Manenti G, Gariboldi M, Fiorino A, Zanesi N, Pierotti MA, Dragani TA (1997). Genetic mapping of lung cancer modifier loci specifically affecting tumor initiation and progression. Cancer Research.

